# 2070. Outbreak of Crimean-Congo Hemorrhagic Fever in Kyzylorda region, Kazakhstan, March-July 2022

**DOI:** 10.1093/ofid/ofad500.140

**Published:** 2023-11-27

**Authors:** Malika Gabdullina, Saya Gazezova, Gulzhan Ayapova, Roberta Horth, Dilyara Nabirova

**Affiliations:** Central Asia Field Epidemiology Training Program, Almaty, Almaty, Kazakhstan; Central Asia Field Epidemiology Training Program, Almaty, Almaty, Kazakhstan; Central Asia Field Epidemiology Training Program, Almaty, Almaty, Kazakhstan; US Centers for Disease Control and Prevention, Dulles, Virginia; CDC Central Asia office, Almaty, Almaty, Kazakhstan

## Abstract

**Background:**

Crimean-Congo Hemorrhagic Fever (CCHF) is a viral tick-borne disease with a high mortality rate. It is endemic in Kazakhstan. The Kyzylorda region reported higher-than-expected cases from March to July 2022 (15 compared to 10 in the previous year). We conducted an investigation to identify additional cases, determine risk factors, and assess CCHF knowledge and practices.

**Methods:**

We conducted a case-control study in July 2022. We defined cases as PCR-confirmed or hospitalized with CCHF symptoms. The control group was made up of regional contacts including neighbors and household members at a 1:2 ratio. We reviewed medical records from April to July. We conducted face-to-face interviews using structured questionnaires. Blood collected from the control group was tested with PCR and ELISA for detection of IgM and IgG antibodies. We also collected ticks from domestic animals for PCR-testing. We used logistic regression to assess factors associated with CCHF.

**Results:**

We identified 38 cases (n=15 confirmed, n=7 probable and n=16 suspected cases) and 71 controls. Cases were mostly male (74%) with mean age of 45 (range 17-81); 27% died. Top patient symptoms (n=38) were weakness (100%), headache (97%), fever (84%) and loss of appetite (55%). Risk factors for CCHF were being male (odds ratio and 95% confidence interval: 5.5, CI: 2.3-13.1), animal husbandry (3.1, 1.3-7.2), crop production (4.0, 1.5-10.5), tick contact (262.5, CI: 31.4-2192.2), tick bite (70.0, 8.8-556.9), contact with tick blood (18.7, 2.2-155.9), ticks on the body or clothing (77.8, 9.8-618.9) and having been in places with high tick risk (15.1, CI: 4.6-49.7). The control group were more likely to have awareness of CCHF, know that they can protect themselves from CCHF, refrained from visiting forests and rural areas, used repellents, and taken protective measures to reduce ticks in domestic animals. Of 55 controls tested: one was IgG positive. Of 163 ticks tested, one was PCR-positive.

**Figure:**
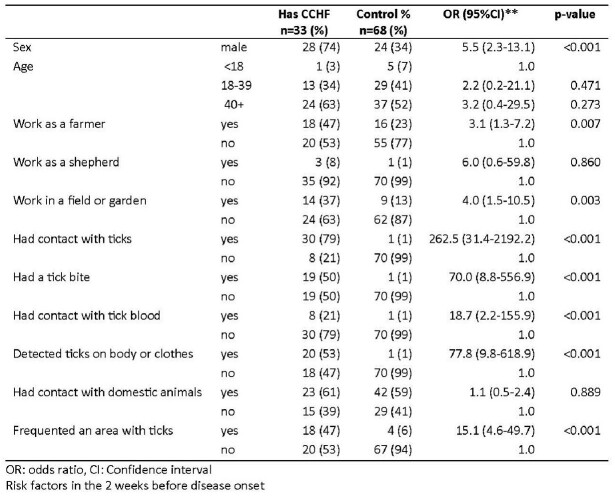

**Figure:**
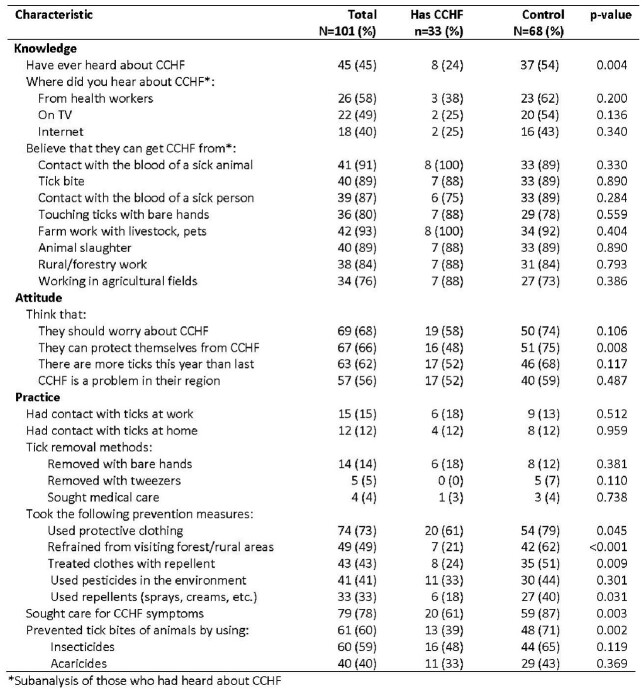

**Conclusion:**

Exposure to activities that are high risk for exposure to ticks increased odds of CCHF. Improved awareness of prevention to exposure from ticks, CCHF associated risk factors, and the importance of seeking early treatment is needed. This can be achieved by increasing population awareness and educating medical providers.

**Disclosures:**

**All Authors**: No reported disclosures

